# ﻿Two new diatom species of the genus *Gomphonemopsis* (Bacillariophyceae) from the coast of China and two new combinations for the genus

**DOI:** 10.3897/phytokeys.239.114018

**Published:** 2024-03-26

**Authors:** Lang Li, Qun-Zhuan Nong, Chang-Ping Chen, Yu-Hang Li, Jun-Xiang Lai

**Affiliations:** 1 Guangxi Key Laboratory of Marine Environmental Science, Guangxi Academy of Marine Sciences, Guangxi Academy of Sciences, Nanning 530007, China Guangxi Academy of Marine Sciences, Guangxi Academy of Sciences Nanning China; 2 Beibu Gulf Marine Industry Research Institute, Fangchenggang 538000, China Beibu Gulf Marine Industry Research Institute Fangchenggang China; 3 School of Resources, Environment and Materials, Guangxi University, Nanning 530004, China Guangxi University Nanning China; 4 School of Life Sciences, Xiamen University, Xiamen 361102, China Xiamen University Xiamen China; 5 Laboratory of Marine Organism Taxonomy and Phylogeny, Qingdao Key Laboratory of Marine Biodiversity and Conservation, Institute of Oceanology, Chinese Academy of Sciences, Qingdao 266071, China Institute of Oceanology, Chinese Academy of Sciences Qingdao China

**Keywords:** Diatom, *Gomphonemopsis*, new species, South China Sea, Yellow Sea

## Abstract

Two new diatom species belonging to the genus *Gomphonemopsis* are described, *Gomphonemopsisnana***sp. nov.** and *Gomphonemopsisgaoi***sp. nov.** These two species were compared in detail with congeners. *Gomphonemopsisnana* is distinguished by its high stria density and small size. This species was found so far to be epiphytic only on the eelgrass collected from Qingdao Bay (Yellow Sea). *Gomphonemopsisgaoi* is characterized by its isopolar valves, simple proximal raphe endings and acutely rounded apices. This taxon was separated from the exoskeleton of marine copepods sampled from the Futian Mangrove Nature Reserve (South China Sea). In addition, two new combinations, *Gomphonemopsisoahuensis* (Hustedt) Lang Li, Yuhang Li & Changping Chen, **comb. nov.** and *Gomphonemopsisplatypus* (Østrup) Lang Li, Yuhang Li & Junxiang Lai, **comb. nov.** are proposed. This study increases the records and knowledge of *Gomphonemopsis* along the coast of China.

## ﻿Introduction

Marine gomphonemoid diatoms are a complex of heteropolar biraphid taxa that are morphologically significantly different from *Gomphonema* Ehrenberg in freshwater environments. The concept was first proposed by Medlin and Round in 1986. Since then, this particular group has included several diatom genera, such as *Cuneolus* Giffen, *Gomphonemopsis* Medlin, *Gomphoseptatum* Medlin, *Pseudogomphonema* Medlin, *Tripterion* R.W.Holmes, S.Nagasawa & H.Takano, *Epiphalaina* R.W.Holmes, S.Nagasawa & H.Takano, *Tursiocola* R.W.Holmes, S.Nagasawa & H.Takano, *Chelonicola* Majewska, De Stefano & Van de Vijver and *Poulinea* Majewska, De Stefano & Van de Vijver (Medlin and Round 1986; Holmes et al. 1993a, 1993b; Denys 1997; Fernandes and Sar 2009; Majewska et al. 2015; Riaux-Gobin et al. 2017). In addition, *Medlinella* Frankovich, M.P.Ashworth & M.J.Sullivan was also considered to belong to marine gomphonemoid diatoms despite of its valvar isopolarity (Frankovich et al. 2016). It worth noting that the habitats of these genera are very special. Most of them are epizoic diatoms on marine vertebrates or epiphytic diatoms on seaweeds and seagrasses. This implies that the gomphonemoid frustules may be related by their epibiotic preference (Medlin 1991).

The genus *Gomphonemopsis* was established and separated from *Gomphonema* based on its morphological features of uniseriate striae, transapically elongated areolae occluded by hymenes, coaxial proximal raphe endings, straight internal raphe fissures, absence of septate valvocopulae and pseudoseptate valves, and lacking stigmata, terminal raphe fissures and basal pore fields (Medlin and Round 1986). Originally *Gomphonemopsis* contained only three species, i.e., *G.exigua* (Kützing) Medlin, *G.pseudexigua* (Simonsen) Medlin and *G.littoralis* (Hendey) Medlin (Medlin and Round 1986). Subsequently, four taxa were transferred to the genus, including *G.domniciae* (Guslakov) Guslakov, *G.obscura* (Krasske) Lange-Bertalot, G.exiguavar.platypus (Østrup) Snoeijs and *G.novo-zelandicum* (Booth) M.A.Harper (Guslakov et al. 1992; Lange-Bertalot et al. 1996; Snoeijs and Balashova 1998; Harper et al. 2012). Recently, three new *Gomphonemopsis* species had also been described, viz., *G.lindae* Witkowski, Metzeltin & Lange-Bertalot, *G.ligowskii* Al-Handal & E.W.Thomas and *G.sieminskae* Krzywda, Gastineau, C.X.Zhou & Witkowski (Metzeltin and Witkowski 1996; Al-Handal et al. 2018; Krzywda et al. 2019). So far, all members of *Gomphonemopsis* have been found in marine or brackish waters. Most of them are distributed in temperate regions (Al-Handal et al. 2018; Krzywda et al. 2019).

Up to now, there are four species of *Gomphonemopsis* reported in China (Li et al. 2005; Cheng and Gao 2013; Sun 2013; Krzywda et al. 2019). In this paper, we report two new *Gomphonemopsis* species sampled from the coasts of the Yellow Sea and the South China Sea and make two new combinations. Detailed morphological descriptions are presented by using light microscopy (LM) and scanning electron microscopy (SEM). Also, similar taxa are compared and information on their ecology is discussed.

## ﻿Materials and methods

Samples were collected at the Qingdao Bay (36°3'33.45"N, 120°18'56.26"E), Qingdao City, the Yellow Sea on 11 October 2022 and at the No. 3 fishing pond (22°31'28.11"N, 114°0'41.37"E) in the Futian Mangrove Nature Reserve, the South China Sea on 14 November 2016. Qingdao Bay is situated in the south of Qingdao City, which lies in the north temperate monsoon zone. This is an open gulf with a natural eelgrass (*Zosteramarina* Linnaeus) bed. The average water depth of Qingdao Bay is about 3.50 m, and the tides here are semidiurnal with an average tidal range of about 2.78 m (Xu et al. 2022). Futian Mangrove Nature Reserve is located in the northeast of Shenzhen Bay. The mean annual air temperature of this location is 23.0 °C (Li et al. 2015). The tides in Shenzhen Bay are also semidiurnal, with an average range of 1.90 m (Gao et al. 2018). Several fishing ponds are present in the mangrove reserve, and the No. 3 fishing pond is connected to Shenzhen Bay through a sluice.

At the site of Qingdao Bay, samples of *Z.marina* were collected by hand at low tide. The eelgrasses were sealed into a Ziploc bag and brought back to the laboratory for further processing. Simultaneously, the temperature and salinity *in situ* were measured with a thermometer and a refractometer (RHS-10ATC), respectively. In the mangrove reserve, samples of marine copepods were taken with a hand net (166 μm) from the No. 3 fishing pond at high tide. Copepods were collected from the bottom of the net and preserved in 5% seawater formalin immediately. Measurements of water temperature and salinity were performed using a U-5000 multi-parameter meter (Horiba, Japan).

Upon return to the laboratory, both samples of eelgrasses and copepods were gently washed with filtered (0.45 μm) seawater for removal of detritus and free microalgae. Diatom cells were separated from host tissues by treating with ultrasound at 300 W for 25 s (Li et al. 2020a, 2020b). They were then acidized with concentrated HCl (36%–38%) at 100 °C for 20 min, followed by rinsing with distilled water to reach neutral pH. For light microscopy (LM) observation, cleaned materials were dried onto coverslips and permanently mounted in Naphrax or Mountmedia. Slides were examined with a Zeiss Imager Z2 (Carl Zeiss, Germany) equipped with differential interference contrast (DIC) and an Olympus BX51 (Olympus, Japan) fitted with phase contrast optics. For scanning electron microscopy (SEM) observation, diatom suspensions were fixed on aluminum stubs after airdrying. Ultrastructural analysis was carried out with a JSM-6390LV (JEOL, Japan) and a Hitachi S-4800 (Hitachi, Japan).

Terminology follows Medlin and Round (1986), Round et al. (1990), Al-Handal et al. (2018) and Krzywda et al. (2019). Because the LM images are not detailed enough to illustrate the morphology, we assigned a SEM image as the iconotype for each species. The term “iconotype” means an icon of the type, which is the most representative illustration of the protologue (Jahn and Kusber 2009). For comparison, SEM illustrations from the literature are cited in Table 1.

**Table 1.** Comparison of measurements and habitats among *Gomphonemopsis* species, modified from Krzywda et al. (2019).

Species	Length (μm)	Width (μm)	Striae (10 μm)	Habitat	References (including SEM documentation if available)
*G.nana*	4.0–7.4	1.1–1.5	26–30	Marine, epiphytic	This paper
*G.gaoi*	28.5–30.5	4.0–5.0	24–26	Brackish, epizoic	This paper
*G.oahuensis*	26	3–4	25	Freshwater	Hustedt 1942
*G.domniciae*	6–8	1.7–2.5	10–18	Marine to brackish, epiphytic and epilithic	Guslakov 1981; Guslakov et al. 1992
*G.littoralis*	14–22	2–3	16–19	Marine, epiphytic	Medlin and Round 1986: SEM figs 52–54
*G.pseudexigua*	3.5–15.0	1.5–2.5	18–22	Brackish, epiphytic	Medlin and Round 1986: SEM figs 48–51
*G.exigua*	9–34	2–6	16–30	Marine and brackish, epiphytic	Medlin and Round 1986: SEM figs 39–45
*G.platypus*	9.5–24	3–4.5	17.5–21	Marine and brackish, epiphytic	Snoeijs and Balasova 1998: SEM fig. 443
*G.lindae*	16.0–18.5	2.5–3.0	18–24	Marine, benthic	Metzeltin and Witkowski 1996: SEM pl. 79: fig. 3, pl. 92: figs 3, 4
*G.ligowskii*	11–17	1.5–2.5	11–14	Marine, epiphytic	Al-Handal et al. 2018: SEM figs 18–22
*G.obscura*	10–17	2–3	16	Marine to brackish, epiphytic	Lange-Bertalot et al. 1996
*G.novozelandicum*	12–35	2–3	20–22	Marine, epiphytic	Booth 1984: SEM fig. 4
*G.sieminskae*	9–18	2.0–2.5	18–22	Brackish, epiphytic	Krzywda et al. 2019: SEM fig. 2C’–G’

## ﻿Results

### ﻿ Gomphonemopsis nana

Taxon classificationPlantaeCymbellalesRhoicospheniaceae

Lang Li, Yuhang Li & Junxiang Lai sp. nov.

38DDCB21-34E2-50C7-8DB0-705066E19F2F

Fig. 1A–P

#### Type materials.

***Holotype*.** Slide MBMCAS286906 deposited in the Marine Biological Museum, Chinese Academy of Sciences (MBMCAS), Qingdao, China.

**Figure 1.**
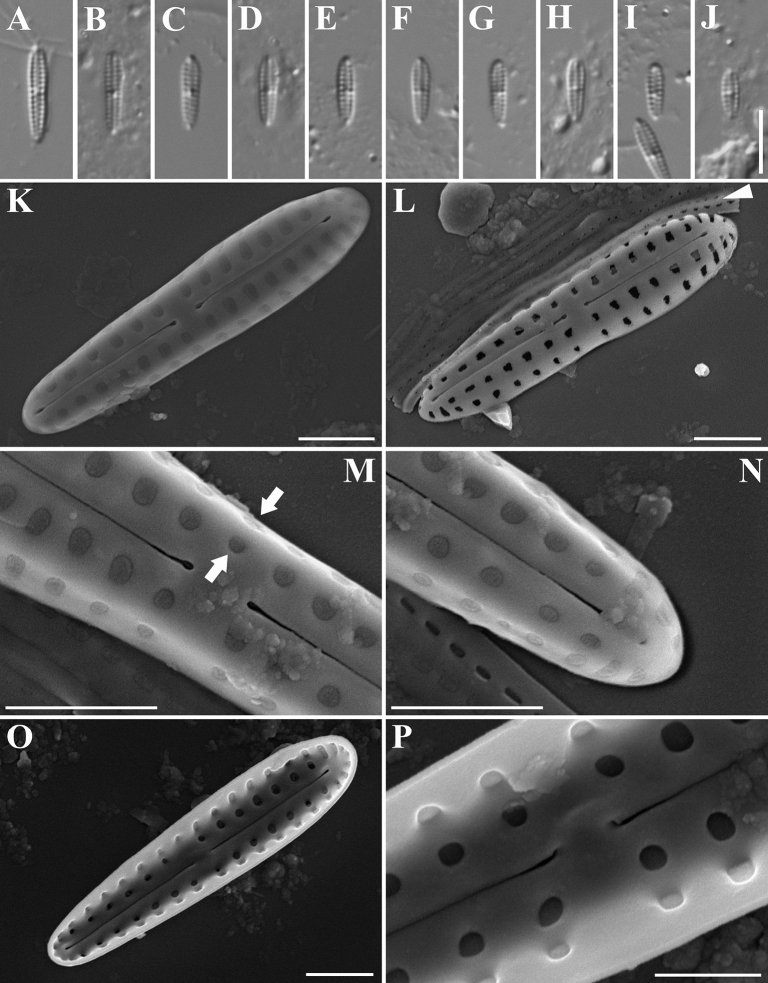
*Gomphonemopsisnana***A–J** light micrographs (differential interference contrast, DIC) **K–P** scanning electron micrographs **K** external view of an entire valve with hymenes covering areolae, iconotype specimen **L** external view of an entire valve without hymenes covering areolae, note the girdle bands perforated with two rows of pores (white arrowhead) **M** external detail of the central area, note the presence of two areolae on the primary side (white arrows) **N** external detail of the foot pole **O** internal view of an entire valve **P** internal detail of the central area. Scale bars: 5 μm (**A–J**); 1 μm (**K–O**); 0.5 μm (**P**).

***Iconotype*.** Fig. 1K.

#### Type locality.

Qingdao Bay, Qingdao City, the Yellow Sea (36°3'33.45"N, 120°18'56.26"E). Collected from the blades of seagrass *Zosteramarina* by Lang Li, 11 October 2022.

#### Description.

***LM*** (Fig. 1A–J). Valves linear, heteropolar with obtusely rounded head pole and sub-acutely rounded foot pole, 4.0–7.4 μm long, 1.1–1.5 μm wide. Axial area very narrow. Raphe indistinguishable in LM. Central area hyaline, extended transapically, or occasionally asymmetrical because of the presence of a stria on primary side of the valve (Fig. 1A). Transapical striae sub-parallel throughout, except slightly radiate at apices, 26–30 in 10 μm.

***SEM*** (Fig. 1K–P). Externally, each stria composed of two elongate to round areolae, one on the valve face, the other on the mantle. A row of areolae presented around apices (Fig. 1K). Areolae occluded by hymenes and becoming smaller towards the foot pole (Fig. 1K). Raphe central, more or less straight (Fig. 1K, L). Proximal raphe endings expanded, pore-like, and deflected in the same direction (Fig. 1K, M). Distal raphe endings slightly expanded and terminating on the valve face (Fig. 1K, L). Central area expanded transapically to the valve margin, but two areolae occasionally present on primary side (Fig. 1M, white arrow). Girdle bands perforated with a double row of pores (Fig. 1L, white arrowhead). Internally, areolae smaller and rounder than external ones (Fig. 1O). Central area slightly elevated. Proximal raphe endings bent to the same side (Fig. 1P) distal raphe endings terminate in small helictoglossae (Fig. 1O).

#### Etymology.

The Latin adjective *nana* refers to the tiny dimensions of the new species as compared to other *Gomphonemopsis* species.

#### Distribution and ecology.

*Gomphonemopsisnana* is an epiphytic species known only from the type locality, where it occurs mainly in the low intertidal region at a temperature of 23.3 °C. The water salinity here was about 30 psu during sampling. Other species that were observed in the same sample include *Amphora* spp., *Navicula* spp., *Nitzschia* spp., *G.exigua* (Kützing) Medlin, *Licmophoracalifornica* Grunow, *Tabulariaparva* (Kützing) D.M.Williams & Round, *T.fasciculata* (C.Agardh) D.M.Williams & Round, *Berkeleyarutilans* (Trentepohl ex Roth) Grunow, *Cocconeisscutellum* Ehrenberg and *Seminavisrobusta* Danielidis & D.G.Mann.

#### PhycoBank registration.

http://phycobank.org/104208.

### ﻿ Gomphonemopsis gaoi

Taxon classificationPlantaeCymbellalesRhoicospheniaceae

Lang Li, Changping Chen & Junxiang Lai sp. nov.

EDFB28B5-27BE-59F1-ACAE-AFE8D1BF1D02

Fig. 2A–P

- Gomphonemopsis aff. G.exigua in Lange and Tiffany 2002, p. 198, fig. 74.

#### Type materials.

***Holotype*.** Slide SZIII161114 deposited in Biology Department Herbarium, Xiamen University (AU), Xiamen, China.

**Figure 2.**
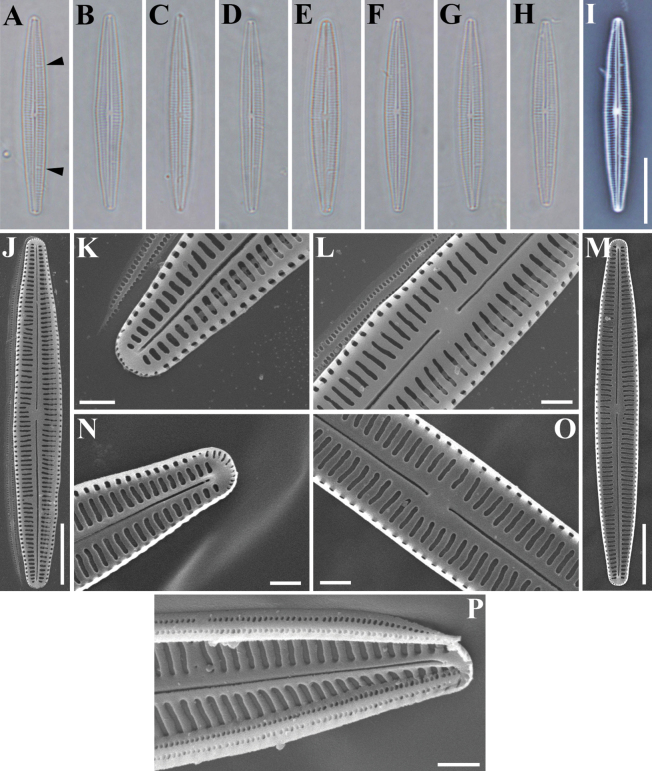
*Gomphonemopsisgaoi***A–I** light micrographs (**I** phase contrast), note the Voigt faults on the secondary side (black arrowheads) **J–P** scanning electron micrographs **J** external view of an entire valve, iconotype specimen **K** external detail of the apex, showing the slit-like pores **L** external detail of the central area **M** internal view of an entire valve **N** internal detail of the apex, showing the slit-like pores **O** internal detail of the central area **P** valvocopula with two rows of subcircular poroids. Scale bars: 10 μm (**A–I**); 5 μm (**J**, **M**); 1 μm (**K, L, N–P**).

***Iconotype*.** Fig. 2J.

#### Type locality.

No. 3 fishing pond, Futian Mangrove Nature Reserve, the South China Sea (22°31'28.11"N, 114°0'41.37"E). Separated from the exoskeleton of marine copepods by Lang Li, 14 November 2016.

#### Description.

***LM*** (Fig. 2A–I). Valves narrowly lanceolate, isopolar with acutely rounded apices, 28.5–30.5 μm long, 4.0–5.0 μm wide. Primary and secondary sides can be easily distinguished because of the obvious interruptions in the stria pattern, which are termed “Voigt faults” (Fig. 2A, black arrowheads). Axial area linear and very narrow, widening towards valve centre. Central area small, sometimes slightly wider on the primary side than the secondary side. Raphe straight with distant simple proximal endings. Striae uniseriate, parallel in the middle and slightly radiate near apices, 24–26 in 10 μm.

***SEM*** (Fig. 2J–P). Valve face flat, with a clear transition to mantle (Fig. 2J). Each of striae comprised of one narrow, elongated areola on valve face and one oblong areola on the mantle (Fig. 2J–L). Valve mantle relatively shallow, with 10–14 radiated slits at apices (Fig. 2K, L). Axial area distinct, forming a narrow, lanceolate hyaline zone and becoming wider in the central area (Fig. 2J). Central area transversely expanded, surrounded by irregularly shortened striae (Fig. 2L). Raphe filiform, composed of two coaxial branches of equal length (Fig. 2J). Both proximal and distal endings almost straight, not enlarged (Fig. 2K, L). Internally, proximal raphe endings small, slightly deflected towards the primary valve side (Fig. 2M, O), whereas distal endings terminate as weakly developed helictoglossae (Fig. 2N). Valvocopula open, possessing two parallel rows of subcircular poroids (Fig. 2P).

#### Etymology.

The epithet honours Prof. Yahui Gao (Xiamen University, China), in recognition of his contributions to marine diatom taxonomy research in China.

#### Distribution and ecology.

In addition to the type locality, *Gomphonemopsisgaoi* may also be distributed in the Salton Sea of the United States (Lange and Tiffany 2002: fig. 74). This taxon is an epizoic diatom on marine copepods. Water temperature of the sampling site was 27.7 °C, and salinity was 12 psu.

#### PhycoBank registration.

http://phycobank.org/104209.

## ﻿Discussion

*Gomphonemopsisnana* sp. nov. possesses heteropolar valves, rounded poles, straight raphe and uniseriate striae consisting of two hymenate areolae but lacks stigmata, terminal raphe fissures, basal pore fields, pseudosepta on the valves and septa on the valvocopulae. All these features justify assigning this new species to the genus *Gomphonemopsis* (Medlin and Round 1986). *G.nana* shares similar stria density with *G.exigua* (Table 1, modified from Krzywda et al. 2019). In addition, both have a row of areolae extending along the whole mantle. However, *G.nana* differs from *G.exigua* by having a wide central area expanding laterally to the valve margin, round to oblong areolae (vs. narrow elongate areolae in *G.exigua*), and a smaller cell (4.0–7.4 µm vs. 9–34 µm). As for other species within the genus, all of them display much lower stria densities than *G.nana*, and most of them have larger cell sizes (Table 1).

*Gomphonemopsisgaoi* sp. nov. also has all the features typical for the genus *Gomphonemopsis* except for its isopolar valves. The taxonomic value of polarity is still under debate (Sabbe et al. 2001). Moreover, in the marine gomphonemoid diatom genus *Tursiocola*, both heteropolar and isopolar species exist (Denys 1997; Frankovich et al. 2015). After assessing the questionable characters, we assigned this species to the genus *Gomphonemopsis*. Despite the difference in valve symmetry, *Gomphonemopsisgaoi* closely resembles *Gomphonemopsisexigua*. Both species have slit-like areolae, narrow axial areas, small central areas, and overlapping valve dimensions and stria densities. However, *Gomphonemopsisgaoi* can be distinguished by its simple proximal raphe endings (vs. pore-like proximal endings), distinctive Voigt faults (vs. lacking Voigt faults) and small slits at both apices (vs. only present at the foot pole). On the other hand, *Gomphonemopsisgaoi* is most similar to *Gomphospheniaoahuensis* (Hustedt) Lange-Bertalot, a freshwater diatom species with isopolar valves and slit-like areolae as well. But there are still some subtle differences between the two species: in *Gomphonemopsisgaoi*, the valve apices are acutely rounded and no T-shaped fissures can be seen at the distal raphe endings, whereas in *Gomphospheniaoahuensis* the valve apices are capitate and the distal raphe endings terminate in T-shaped depressions (Hustedt 1942; Simonsen 1987; Moser et al. 1998).

Lange-Bertalot established a subgenus Costericardia Lange-Bertalot under the genus *Gomphosphenia* Lange-Bertalot to accommodate the isopolar and naviculoid species, i.e., *Gomphospheniaoahuensis* (Moser et al. 1998). However, *Gomphospheniaoahuensis* lacks the diagnostic feature of the genus *Gomphosphenia*, namely anchor or T-shaped internal proximal raphe endings. In addition, as in *Gomphonemopsisgaoi*, *Gomphospheniaoahuensis* also has all the features of *Gomphonemopsis*, except for the polarity. Therefore, we propose the transfer of *Gomphospheniaoahuensis* to *Gomphonemopsis.* An alternative option would be to establish a new genus to accommodate *Gomphonemopsisgaoi* and *Gomphospheniaoahuensis*, because their valves are isopolar rather than heteropolar. However, in the absence of supporting molecular data, we refrain from doing so.

### ﻿ Gomphonemopsis oahuensis

Taxon classificationPlantaeCymbellalesRhoicospheniaceae

(Hustedt) Lang Li, Yuhang Li & Changping Chen comb. nov.

71205A2F-2103-59B1-82DD-E0627CDE9597

Cymbella oahuensis Hustedt 1942. Internationale Revue der gesamten Hydrobiologie und Hydrographie 42 (1/3): p. 98, figs 193–195. Lectotype: designated by Simonsen (1987, p. 282). BRM 163/65, illustrated as pl. 416, figs 4–8. Basionym.
Gomphosphenia oahuensis (Hustedt) Lange-Bertalot in Moser, Lange-Bertalot and Metzeltin 1998, p. 42, pl. 5, figs 6–8, pl. 53, figs 1–9. Synonyms.
Navicula oahuensis (Hustedt) Krammer in Krammer and Lange-Bertalot 1985, p. 83.

#### PhycoBank registration.

http://phycobank.org/104211.

#### Notes.

Gomphonemopsisexiguavar.platypus was originally described from Bornholm, Denmark as *Gomphonemaplatypus* Østrup. Subsequently, Krammer and Lange-Bertalot (1985) reclassified this taxon as a variety of *Gomphonemaexiguum*. Snoeijs transferred it to *Gomphonemopsis* (Snoeijs and Balashova 1998). Despite sharing a similar size dimension and stria density with the nominate variety (Medlin and Round 1986; Snoeijs and Balashova 1998), it has a unique widened foot pole differing from other congeners (Snoeijs and Balashova 1998). Therefore, we suggest elevating Gomphonemopsisexiguavar.platypus to the species level.

### ﻿ Gomphonemopsis platypus

Taxon classificationPlantaeCymbellalesRhoicospheniaceae

(Østrup) Lang Li, Yuhang Li & Junxiang Lai comb. nov.

B11FA152-A728-5E30-85CA-55AF647FCEF1

Gomphonema platypus Østrup 1910. Danske Diatoméer, p. 65, pl. II, fig. 49. Basionym.
Gomphonemopsis exigua var. platypus (Østrup) Snoeijs in Snoeijs and Balashova 1998, p. 55, fig. 443. Synonyms.
Gomphonema exiguum var. platypus (Østrup) Lange-Bertalot in Krammer and Lange-Bertalot 1985, p. 47.

#### PhycoBank registration.

http://phycobank.org/104420.

##### ﻿Dichotomous key to distinguish the *Gomphonemopsis* species

In order to facilitate the identification of the *Gomphonemopsis* species, a dichotomous key to all known species is presented as follows:

**Table:** 

1	Valves isopolar	**2**
–	Valves heteropolar	**3**
2	Apices capitate	***G.oahuensis***
–	Apices acutely rounded	***G.gaoi***
3	Valves clavate with widened foot pole	***G.platypus***
–	Valves linear to lanceolate	**4**
4	Central area small	***G.exigua***
–	Central area wide or asymmetrical	**5**
5	Striae 26–30 in 10 μm	***G.nana***
–	Striae ≤ 24 in 10 μm	**6**
6	Areolae round	**7**
–	Areolae elongate or round near poles	**8**
7	Mantle areolae only extending along the wider part of valve	***G.ligowskii***
–	Mantle areolae extending along the whole mantle	***G.littoralis***
8	Striae ≤ 18 in 10 μm	**9**
–	Striae ≥ 18 in 10 μm	**10**
9	Valves 10–17 μm long, 2–3 μm wide; striae 16 in 10 μm	***G.obscura***
–	Valves 6–8 μm long, 1.7–2.5 μm wide; striae 10–18 in 10 μm	***G.domniciae***
10	Central area extending to valve/mantle junction	**11**
–	Central area extending to valve margin	**12**
11	Transapical striae divided the into two parts	***G.sieminskae***
–	Transapical striae not divided the into two parts	***G.pseudexigua***
12	A row of small slits around the foot pole	***G.lindae***
–	One or two pores around the foot pole	***G.novozelandicum***

To date, the genus *Gomphonemopsis* contains thirteen diatom species, six of which have been reported in China seas. This genus may have a wider distribution in the marine coastal waters of subtropical to Polar regions, with the exception of *G.oahuensis*, which lives in tropical freshwater environments. According to Table 1, *Gomphonemopsis* exhibits diverse habitat preferences. Most species are epiphytic on seaweeds and seagrasses, whereas, interestingly, *G.gaoi* “chooses” copepods as its hosts in this study. This may be not the first report of epizoic *modus vivendi* in *G.gaoi*. Lange and Tiffany (2002) found that this species could attach to both green algae and the stalk of the ciliate, but they couldn’t determine whether the ciliate was its strict host. Hence, further ecological studies are needed to reveal the habitats of *G.gaoi* and other species within the genus.

## Supplementary Material

XML Treatment for Gomphonemopsis nana

XML Treatment for Gomphonemopsis gaoi

XML Treatment for Gomphonemopsis oahuensis

XML Treatment for Gomphonemopsis platypus

## ﻿Additional information

### Author ORCIDs

Lang Li https://orcid.org/0000-0001-5649-1097

Qun-Zhuan Nong https://orcid.org/0009-0004-6646-1528

Chang-Ping Chen https://orcid.org/0000-0002-3332-2458

Yu-Hang Li https://orcid.org/0000-0002-5546-1774

Jun-Xiang Lai https://orcid.org/0000-0002-7139-1454

### Data availability

All of the data that support the findings of this study are available in the main text.
